# Survival of *Streptococcus equi* subsp. *zooepidemicus* on environmental samples is affected by material type and incubation temperature

**DOI:** 10.1007/s11259-023-10095-0

**Published:** 2023-04-17

**Authors:** Sulove Koirala, Carolina Pantuzza, Matheus de O. Costa

**Affiliations:** 1 Large Animal Clinical Sciences, Western College of Veterinary Medicine, 52 campus drive, Saskatoon, SK Canada; 2 Escola de Veterinária, Universidade Federal de Minas Gerais, Belo Horizonte, Brazil; 3 Population Clinical Sciences, Faculty of Veterinary Medicine, Utrecht University, Utrecht, The Netherlands

**Keywords:** Zooepidemicus, swine, sepsis, sudden death, disease, enviroment, persistence, contamination

## Abstract

*Streptococcus equi* subsp. *zooepidemicus* is an opportunistic pathogen associated with disease in a range of domestic and wild animals. Despite its importance, very limited data is available on its survival and persistence on the environment. The goal of this study was to evaluate survival of *S. zooepidemicus* under ideal culture conditions and farm-like setting, in various surface types. Rubber, plastic, wood, and concrete samples were sterilized and inoculated with 10^9^ CFU/mL of *S. zooepidemicus* with or without feces, and cultured under ideal conditions (37 °C, 5% CO_2_) or farm-like settings (20^o^C on air) for a maximum period of 25 days (n = 3/material/environment/feces-group/time-point). Under ideal conditions without feces, the bacterium survived for up to 17 days on plastic and rubber surfaces, 4 days on wood and less than 1 day on concrete (*P* < 0.05 between materials). Samples under ideal conditions with feces and farm-like settings without feces were negative by day 1 post-inoculation, regardless of the surface material used. Wood and concrete allowed *S. zooepidemicus* persistence for up to 3 days under farm-like settings when feces were present. This data suggests that environmental persistence of *S. zooepidemicus* is affected by surface type and incubation temperature.

## Introduction

*Streptococcus equi* subsp. *zooepidemicus* (*S. zooepidemicus*) is linked to disease in multiple domestic animal species, often concurrent with severe outbreaks leading to increased mortality (Bade et al. 2009; Cebra et al. 2000; Costa and Lage 2020; FitzGerald et al. 2017; Lamm et al. 2010; Pisoni et al. 2009; Priestnall and Erles 2011; Soedarmanto et al. 1996). It has also been related to zoonotic disease in immunosuppressed or elderly patients (Kerdsin et al. 2021; Kim et al. 2022; Klapa et al. 2021; Matsubayashi et al. 2021; Torres et al. 2018). This Gram-positive, Lancefield group C, facultative anaerobe is commonly identified in the upper respiratory or reproductive tract mucosa of adult domestic animals without disease (Kernaghan et al. 2012; Li et al. 2021). In 2019 *S. zooepidemicus* emerged in North America as cause of mass mortality and increased abortion rates in pigs (Costa and Lage 2020; Sitthicharoenchai et al. 2020). Clinically, the disease was undistinguishable from African Swine Fever, an exotic disease not yet identifed in the United States of America or Canada with colossal impacts to the pork industry, as evidenced in a recent outbreak in China (Costa et al. 2022; Wang et al. 2018).

*S. zooepidemicus* is a subspecies of *S. equi*, alongside *S. equi* subsp. *equi* (*S. equi)*, the causative agent of strangles in horses, with greater than 98% DNA homology between the two subspecies (Timoney 2004). The impact of *S. equi* to the equine industry is remarkable, and it also has zoonotic potential. Contrary to *S. zooepidemicus*, a commercial vaccine is available for horses in selected countries, aiding in disease control and prevention (Robinson et al. 2020). While published data on *S. equi* environmental persistence is limited to 2 studies (Durham et al. 2018; Weese et al. 2009), to the best of our knowledge there is no data on the persistence of *S. zooepidemicus* on the environment other than composted equine litter (Poulin et al. 2015). This evidences an obvious knowledge gap on *S. zooepidemicus* epidemiology and the potential for spreading, via contaminated surfaces and environment.

The goal of this study was to investigate the survival period of *Streptococcus equi* subsp. *zooepidemicus* on surfaces present in commercial swine operations under ideal culture conditions and farm-like settings.

## Methods

### Preparation of inoculum

A pure culture of *S. zooepidemicus* ST-194 was used as inoculum (Costa and Lage 2020). This isolate was resistant to lincomycin, neomycin and tetracycline, based on the Kirby-Bauer disk diffusion method. Zones of growth inhibition were interpreted according to Clinical and Laboratory Standards Institute (CLSI) guidelines (Lewis 2022). Culture was performed using columbia colistin nalidixic acid (CNA) blood agar plates with 5% sheep blood (Becton, Dickinson Co., Maryland, USA). The plate was incubated for 24 h at 37 °C in an incubator gassed with 5% CO_2_. Using a sterile loop, a single colony was scrapped and transferred to a 50 mL conical tube (Corning Life Sciences, New York, USA) containing brain heart infusion broth (BHI) broth. This was incubated for 8 h in the conditions described above. Optical density (OD) of the broth was measured using a spectrophotometer (NanoDrop 2000, Thermo Fisher Scientific, Massachusetts, USA) at 600 nm (OD 600) to achieve a final concentration of 1.4 × 10^9^ CFU/mL. The inoculum was plated in a CNA agar plate prior to inoculation to confirm viability.

### Preparation of surface samples

The surface area of all materials tested was 1 cm^2^. Rubber samples were melamine free (VWR, Pennsylvania, USA, Cat. # 59580-069). Polyethylene terephthalate (PET) plastic from household bottles were used as plastic surface. Northern white birch plank (Fisher Scientific, New Hampshire, USA, Cat. # S80332) was used to simulate wood surface. Commercial ready-mixed concrete (DAP, Maryland, USA) was used by setting it on a silicon tray, then removing the solidified material prior to use. All surface samples were autoclaved (121^o^C at 15 psi for 30 min) prior to inoculation.

### Preparation of fecal samples

To simulate farm-like settings where organic matter is often present, a fresh fecal sample was collected from an 8 weeks-old, commercial crossbred castrated barrow free of any clinical signs suggestive of disease, including *S. zooepidemicus*. The sample was obtained following digital stimulation, and did not come into contact with the floor or any other surface. It was stored at -20^o^C until autoclaved at 121^o^C for 30 min and 15 psi. Autoclaved samples were stored at -20^o^C until use.

### Inoculation and incubation of surfaces

Samples were either made of rubber, wood, plastic or concrete (material), and incubated at 37 °C, 5% CO_2_ (ideal conditions) or 20^o^C on air, 70% humidity (farm-like setting, environment), with or without feces (feces-group) over 10 time points (day 0 – within 1 h of inoculation and 1, 2, 3, 4, 7, 9, 13, 17, 20 and 25 days post-inoculation, time-point). A total of 480 surface samples were prepared for inoculation (n = 3/material/environment/feces-group/time-point).

Samples without feces were inoculated using 20 µL of inoculum and spread around the flat surface using sterile pipette tips, in order to cover the entire surface. Autoclaved feces were mixed at a ratio of 2:1 v/v with the inoculum (20 µL) to achieve a pasty consistency. This mixture was gently spread on top of the samples using a sterile 10 µL loop to cover the entire surface. Each sample was kept in a single well of 6-well tissue culture plate (Fisher Scientific, New Hampshire, USA,).

### Bacterial viability assessment

At any given time point, 3 samples/material/environment/feces-group were assessed for viability of the inoculum. First, 8 mL of BHI broth was added to each well, covering the entire inoculated material. Broth-immersed surface samples were incubated for 12 h at 37 °C, 5% CO_2_, and a 10 µL aliquot was plated in CNA blood agar for further incubation at 37 °C, 5% CO_2_ for 24 h. Samples were considered positive if turbidity was observed in the broth sample and beta-haemolytic, mucoid, transparent colonies were visible in the agar plates following incubation. Sampling stopped for a given group if two consecutive samplings resulted in no growth in both broth and agar plates.

### Statistical analyses

Differences between bacterial survival period on different materials within culture conditions and feces group was evaluated using the Mantel-Cox test (SPSS v23, IBM, Waltham, MA).

## Results

**Fig. 1:**
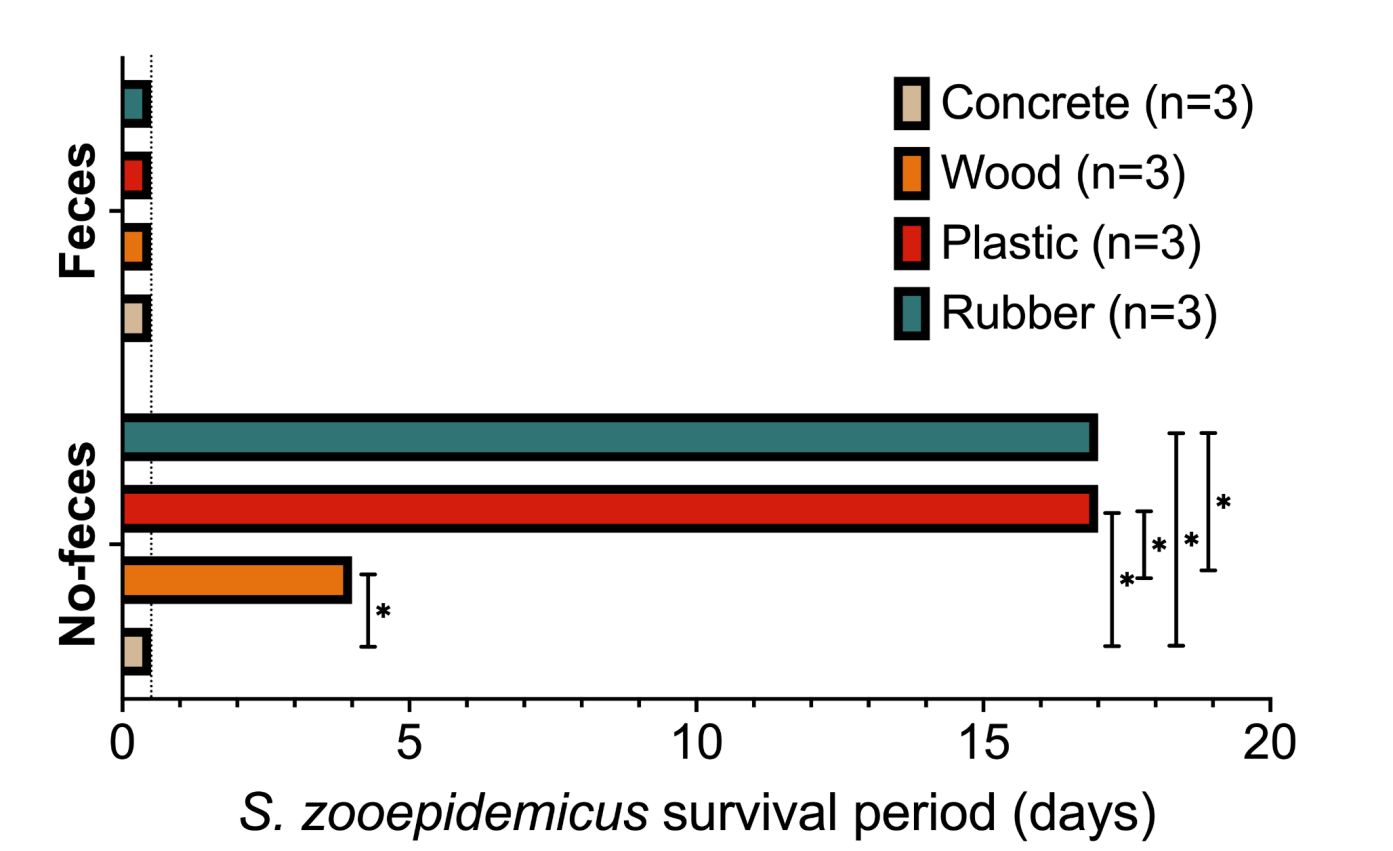
*S. zooepidemicus* survival period under ideal culture conditions (37 °C, 5% CO_2_) on different surface materials with or without the presence of feces. Survival period is reported as the last day where a positive bacterial culture was obtained. Dotted line depicts post-inoculation control (1-hour). *- Significant difference between materials within feces group, as per Mantel-Cox test

Viable bacteria were present in all groups immediately after inoculation. Plastic and rubber samples incubated at ideal conditions for *S. zooepidemicus* growth without feces enabled bacterial survival for up to 17 days, inclusive. This survival period was significantly different from wood and concrete surfaces (*P =* 0.004 and *P =* 0.001, respectively). Survival on wood was also significantly increased when compared to concrete (4 days and less than 1 day, respectively, *P* = 0.02). The addition of feces resulted in bacterial inactivation within 1 day of inoculation. A summary of these data is shown in Fig. 1.

**Fig. 2:**
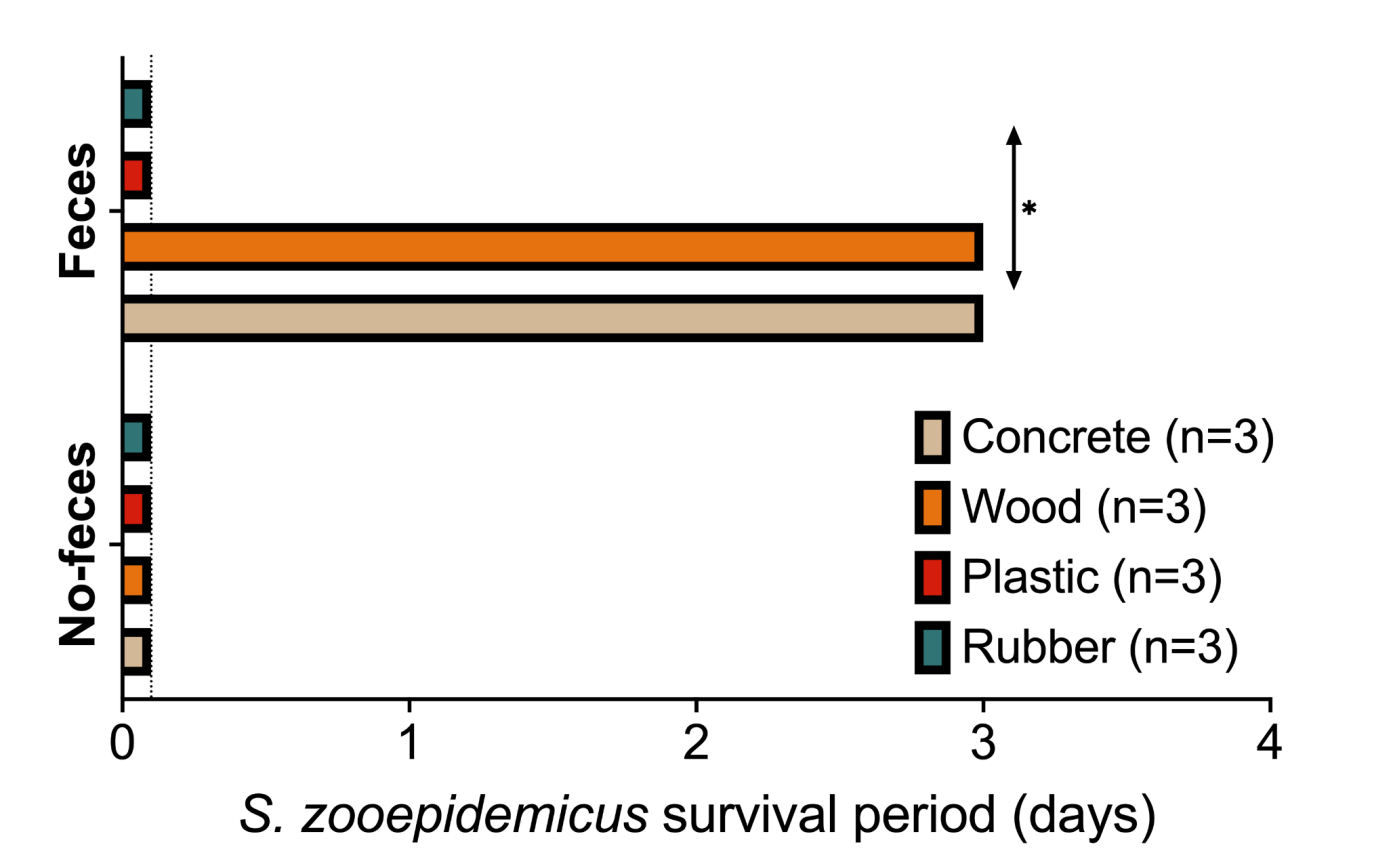
*S. zooepidemicus* survival period under farm-like conditions (20^o^C on air, 30% humidity) on different surface materials with or without the presence of feces. Survival period is reported as the last day where a positive bacterial culture was obtained. Dotted line depicts post-inoculation control (1-hour). *- Significant difference between materials within feces group, as per Mantel-Cox test

When samples were kept at 20^o^C the absence of feces resulted in no bacterial survival for longer than 24 h (Fig. 2). In contrast, the presence of feces extended *S. zooepidemicus* survival for up to 3 days in concrete and surface samples, when compared to rubber or plastic (*P* = 0.04). Plastic and rubber surfaces inoculated with feces were negative by 1 day post-inoculation.

## Discussion

The data presented here evidenced that the survival of *S. zooepidemicus* in the environment is affected by the culture conditions, the presence of feces and the material type of the inoculated surface. Interestingly, under ideal culture conditions (37 °C, 5% CO_2_) the presence of feces inhibited bacterial growth, regardless of the surface material. The opposite was observed at room temperature, where surfaces inoculated with feces and *S. zooepidemicus* resulted in longer bacterial survival period. This data may guide the development of future biosecurity and disease elimination protocols, particularly in swine operations.

*S. zooepidemicus* survival in the environment is very poorly studied. A single report investigated its survival in compost of horse litter (Poulin et al. 2015). In that study, autoclaved litter at room temperature (21^o^C-23^o^C) enabled the survival of *S. zooepidemicus* for 5 days. This is similar what we found when inoculated wood and concrete surfaces were incubated with autoclaved swine feces (3 days) at 20^o^C. In parallel, *Streptococcus equi* subsp. *equi* (*S. equi)* is suggested to persist in wet sites for 9 days, and dry sites for 2 days outdoor in the summer. Persistence increased in the winter, for up to 34 days (Durham et al. 2018). A second study reported that *S. equi* could not survive for longer than 3 days on bare wood, painted wood, metal or rubber surfaces during summer and under the exposure to sunlight (which is not present in modern swine barns, that are fully enclosed) (Weese et al. 2009). In contrast to our findings, Weese et al., 2009 did not identify an effect associated with surface type, but equine respiratory mucus showed a degree of protective effect on bacterial survival. It is also noteworthy that both studies using *S. equi* did not use autoclaved surfaces, which may have a potential positive or negative impact on survival of the bacterium of interest. While autoclaving the surfaces does not simulate farm-like settings, it removes any live competitors or inhibitors produced by other microbes, allowing the direct evaluation of *S. zooepidemicus* survival in a best-case scenario. Based on this, our data could be cautiously extrapolated to infer that *S. zooepidemicus* survival for longer than 17 days in a barn is unlikely.

*S. zooepidemicus* is an opportunistic pathogen capable of infecting a wide range of warm and cold-blooded hosts(Abbott et al. 2010; Bade et al. 2009; Cebra et al. 2000; Corpa et al. 2018; Garmyn et al. 2020; Pisoni et al. 2009; Priestnall and Erles 2011; Soedarmanto et al. 1996; Stoughton and Gold 2015). Its importance to livestock is often reported as sporadic outbreaks, controlled following antimicrobial therapy (Pelkonen et al. 2013; Pisoni et al. 2009). However, since 2019, *S. zooepidemicus* emerged as a significant cause of mortality and abortions in swine reared in commercial settings, leading to significant economic loss to producers in North America (Costa and Lage 2020). These outbreaks, described by Costa and Lage, 2020, were associated with a specific sequence type – ST-194, the strain used in this study. Many questions surround this emergence in North American swine farms in the past 3 years, including the missing epidemiological link between the multiple sites affected across North America (Kuchipudi et al. 2021; M. Houben, 2021; Sitthicharoenchai et al., 2020). While direct contact seems to be required for infection of pigs, it is still unclear if *S. zooepidemicus* is able to persist in the environment and potentially become a source for re(infection) of pigs (Costa et al. 2022). Since no efficacious vaccine is available for use in this species, efforts to control this pathogen are largely based on biosecurity and antimicrobial therapy. A recent attempt to eliminate the pathogen by herd depopulation in a commercial swine operation was unsuccessful. Despite vigorous washing, disinfection and 45 days down time prior to repopulating the site with pigs from a herd free *S. zooepidemicus*, naïve animals were diagnosed with the disease weeks following reintroduction (White 2022). In this case, environmental persistence was suggested as one of the potential routes leading to the re-break. While this remains to be clarified in this specific case, our data shows that environmental contamination may not be a key factor here, whereas the presence of pests or other potential live carriers may have played a bigger role.

We understand that the data presented here is not definitive, and further studies looking at *S. zooepidemicus* survival under other conditions are required. However, it provides an initial look at the potential for persistence of this pathogen in areas where active shedding happened. It is difficult to make specific guidelines for the management of cleaning and decontamination protocols of potentially contaminated premises. Nevertheless, under the conditions of modern commercial swine farms, it is reasonable to expect that the environment should not be a source of infection for animals following cleaning and disinfection procedures and if pigs are reared in batches. This is radically different for backyard or small scale, organic premises where sunlight and the weather may directly impact pathogen survival, as well as wildlife may be in contact with infected pigs.

## Conclusion

*S. zooepidemicus* persists for a limited period in an abiotic surface. Under the conditions of this study, the longest period where viable bacterium was detected was for 17 days at 37^o^C on plastic and rubber surfaces without feces. Concrete and wood supported bacterial viability for 3 days at 20^o^C if feces were present, the longest range detected under farm-like settings. Environmental contamination is an unlikely route of pathogen dissemination if biosecurity protocols are in place, but it may happen in unsupervised conditions. Future research on *S. zooepidemicus* viability on colder, winter-like temperatures is warranted to help further understand its epidemiology.

## Data Availability

All the data from this study is available in the manuscript.
